# Humic Acid Therapy Mitigates Estrogen Deficiency-Induced Alveolar Bone Loss and Modulates the RANKL/OPG Balance

**DOI:** 10.3390/biomedicines14061244

**Published:** 2026-05-30

**Authors:** Larissa Vieira Toledo, Maíra Gabrielle de Abreu Ribeiro, Thays Cristina dos Santos, Maria Luiza Nonato Salvador, Natália Oliveira Bertolini, Jaqueline do Carmo Lima Carvalho, Débora Ribeiro Orlando, Rafael Neodini Remedio, Alan Rodrigues Teixeira Machado, Leonardo Barros Dobbss, Stela Márcia Pereira Dourado, Luciano José Pereira, Eric Francelino Andrade

**Affiliations:** 1 Faculty of Health Sciences, Universidade Federal de Lavras (UFLA), Lavras 37200-000, Minas Gerais, Brazil; larissa.toledo3@estudante.ufla.br (L.V.T.); maira.ribeiro@estudante.ufla.br (M.G.d.A.R.); thays.santos1@estudante.ufla.br (T.C.d.S.); maria.salvador@estudante.ufla.br (M.L.N.S.); debora.orlando@ufla.br (D.R.O.); rafael.remedio@ufla.br (R.N.R.); stelapereira@ufla.br (S.M.P.D.); lucianojosepereira@ufla.br (L.J.P.); 2 Department of Physical Education, University Center of Lavras (UNILAVRAS), Lavras 37200-000, Minas Gerais, Brazil; natioliveira.ef@gmail.com; 3 Department of Exact Sciences, Universidade do Estado de Minas Gerais, João Monlevade 35930-314, Minas Gerais, Brazil; jaqueline.z.lima@gmail.com (J.d.C.L.C.); alan.machado@uemg.br (A.R.T.M.); 4 Institute of Agrarian Sciences, Universidade Federal dos Vales do Jequitinhonha e Mucuri (UFVJM), Unaí 38610-000, Minas Gerais, Brazil; leonardo.dobbss@ufvjm.edu.br

**Keywords:** alveolar bone, estrogen depletion, humic substances, bone resorption, natural products

## Abstract

**Background:** Estrogen deficiency negatively affects alveolar bone by disrupting key regulators of bone remodeling. Humic acids (HAs) are natural compounds with recognized antioxidant and anti-inflammatory properties that may attenuate bone resorption. This study investigated the effects of HAs on alveolar bone in an experimental model of estrogen depletion. **Methods:** Female C57BL/6 mice were randomly assigned to four groups: Sham, Sham + HA, ovariectomized (OVX), and OVX + HA. Estrogen deficiency was induced by bilateral ovariectomy. HAs derived from vermicomposted biomass were administered daily by oral gavage (80 mg/kg) for 28 days. At the end of the experimental period, mandibles were collected for structural, mineral, and histological analyses. Bone elemental composition was assessed using scanning electron microscopy coupled with energy-dispersive spectroscopy (SEM/EDS). Alveolar bone loss was evaluated by histomorphometry, while RANKL and osteoprotegerin (OPG) expression were assessed by immunohistochemistry. Osteoclasts were quantified by tartrate-resistant acid phosphatase (TRAP) staining. Data were analyzed using two-way ANOVA followed by Bonferroni’s post hoc test. **Results:** Ovariectomy resulted in reduced calcium and phosphorus content, increased alveolar bone loss, elevated RANKL immunolabeling, increased osteoclast numbers, and a higher RANKL/OPG ratio (*p* < 0.05). HA treatment increased calcium and phosphorus content and attenuated alveolar bone loss in OVX animals (*p* < 0.05). Additionally, HA treatment partially increased OPG expression and reduced the RANKL/OPG ratio (*p* < 0.05), without significantly affecting RANKL immunolabeling or osteoclast numbers. **Conclusions:** HA therapy attenuated alveolar bone resorption in a model of estrogen depletion, possibly associated with modulation of the RANKL/OPG balance.

## 1. Introduction

Estrogen deficiency resulting from menopause promotes an imbalance in bone remodeling, leading to osteopenia and ultimately to osteoporosis [1]. Bone remodeling is primarily regulated by the RANK–RANKL–OPG signaling axis. In this system, the receptor activator of nuclear factor kappa-B ligand (RANKL) binds to its receptor RANK, which is expressed on osteoclasts and their precursors, thereby stimulating osteoclastogenesis and promoting bone resorption [2]. Osteoprotegerin (OPG) is a key regulatory molecule involved in modulating and inhibiting the interaction between RANKL and RANK, thereby protecting against bone demineralization [3]. Among other regulatory factors, OPG expression is stimulated by estradiol, and the decline in estrogen levels during menopause enhances bone resorption [4]. Additionally, the increase in pro-inflammatory mediators observed during menopause promotes osteoclastogenesis, further contributing to the progression of osteoporosis [5].

According to data from the Global Burden of Disease (GBD) Study 2021, low bone mineral density associated with postmenopausal osteoporosis was responsible for 219,552 deaths and 7.76 million disability-adjusted life years [6]. In the same study, it was projected that by 2045, the number of deaths attributable to this factor will exceed 386,000 [6]. In long bones, this condition increases the risk of fractures, leading to impairments in quality of life and increased public healthcare expenditures [7]. In the alveolar bone, menopause-related demineralization promotes the loss of support for teeth and dental prostheses, in addition to aesthetic and functional impairments [8,9]. Therefore, in addition to the systemic effects and those on long bones resulting from estrogen depletion, numerous studies have focused on therapeutic strategies aimed at preserving alveolar bone in order to prevent these adverse outcomes [10,11,12].

Traditional therapies for the treatment of osteoporosis in postmenopausal women include the use of bisphosphonates (e.g., alendronate and risedronate) and hormone replacement therapy, such as selective estrogen receptor modulators, among others [13]. However, these treatments may cause adverse effects, including atypical fractures, gastrointestinal complications, and an increased risk of tumor development [14], which has increased interest in natural product-based adjunctive therapies. Among natural product-based therapies for the treatment of osteoporosis, those containing flavonoids, monoterpenes, triterpenes, and polyphenols are particularly noteworthy, as they primarily act by protecting against oxidative damage and suppressing pro-inflammatory cytokines [15]. In this context, humic acids (HAs) are macromolecules with recognized anti-inflammatory and antioxidant properties [16] that remain poorly investigated for the treatment of postmenopausal osteoporosis.

HAs can be extracted from biomass through different processes and are characterized by a variety of functional groups, including hydrophilic hydroxyl (–OH) moieties and hydrophobic fragments composed of aliphatic chains and aromatic rings [17]. Despite their widespread use in agriculture as fertilizers, these compounds exhibit antitumoral, antiviral, wound-healing, anti-inflammatory, and antioxidant properties [16]. Their anti-inflammatory effects occur through the modulation of leukocyte adhesion and the regulation of key inflammatory signaling pathways, including NF-κB-, MAPK-, PI3K/Akt-, and Toll-like receptor (TLR)-mediated cascades, thereby reducing the expression of endothelial adhesion molecules and the release of oxidative enzymes [18]. Furthermore, the antioxidant properties of HAs are attributed to their ability to regulate reactive oxygen and nitrogen species [18]. In addition to their potential health benefits, these compounds can be obtained from biomass, thereby reducing environmental impact and promoting the circular economy [19].

Regarding biomedical applications, in a previous study published by our group, we demonstrated that, in a preclinical model of periodontitis, treatment with HAs improved the mineral content of the alveolar bone and attenuated alveolar bone resorption [20]. Additionally, in another pioneering study, we observed that HA treatment preserved calcium and phosphorus levels in the femur and modulated the hepatic and renal oxidative profiles in an experimental model of estrogen depletion [21]. Despite these findings, to date, no studies have demonstrated the effects of therapy with these compounds on alveolar bone in a model of estrogen depletion. Therefore, considering that menopause promotes systemic bone resorption and affects alveolar bone remodeling, we aimed to investigate the effects of HA treatment on the characteristics of the mandibular bone in ovariectomized mice.

## 2. Materials and Methods

This study received approval from the Animal Use Ethics Committee of the Federal University of Lavras (CEUA/UFLA; protocol no. 3643011123). All experimental procedures were carried out in accordance with the Guide for the Care and Use of Laboratory Animals and complied with the ARRIVE guidelines. The in vivo experiments were performed between August and September 2024.

### 2.1. Composting, Vermicomposting, Extraction, and Characterization of Humic Acids (HAs)

Vegetal waste from the agricultural production of soybean (*Glycine max* L.) and sorghum (*Sorghum bicolor* (L.) Moench) was coarsely chopped, and along with corn grain flour (*Zea mays* L.) and shredded sugarcane bagasse (*Saccharum officinarum* L.), was composted for 30 days, with mechanical turning performed every 10 days. Subsequently, earthworms (*Eisenia foetida*) were introduced at a density of 50 individuals per kilogram of organic material to initiate vermicomposting, which lasted approximately three months (95 days). Throughout this period, environmental parameters, including humidity, temperature, pH, and aeration, were monitored at three-day intervals. After vermicomposting, the resulting vermicompost was oven-dried at 60 °C for 48 h, passed through a 4 mm mesh sieve, and utilized for the extraction of humic acids (HAs).

The extraction procedure involved treating the vermicompost with a 0.1 mol L^−1^ sodium hydroxide (NaOH) solution at a 1:10 (m/v) ratio under continuous agitation for four hours. The suspension was then centrifuged (15 min at 5000 g) to separate the soluble humic substances, comprising humic acids (HAs) and fulvic acids (FAs), from the insoluble humin fraction. To isolate the HAs from the FAs, the pH of the supernatant was adjusted to between 1.0 and 1.5 using 6 mol L^−1^ hydrochloric acid (HCl), resulting in HA precipitation. The precipitated material was subsequently recovered and repeatedly washed with distilled water until the absence of chloride ions was confirmed by a negative silver nitrate (AgNO_3_) test. The purified HAs were then neutralized to pH 7.0 with 0.01 mol L^−1^ potassium hydroxide (KOH), transferred to dialysis membranes with a molecular weight cut-off of 1000 Da, and dialyzed against distilled water until stabilization of the system’s electrical conductivity (EC). Finally, the dialyzed HAs were frozen and lyophilized for subsequent use. The physicochemical characterization of the HAs employed in this study has been previously reported by our research group [20,22,23].

### 2.2. Animals

A total of 24 healthy female C57BL/6 mice, 12 weeks old and presenting an average initial body mass of 20.8 ± 0.8 g, were utilized. The animals were obtained from the Laboratory Animal Breeding Center (NUCAL) of the Federal University of São João del-Rey, located in Minas Gerais, Brazil. Throughout the experimental protocol, the mice were maintained in an animal facility under controlled environmental conditions (22 ± 2 °C, relative humidity of 45 ± 15%, and a 12 h light/dark cycle). The animals were housed in polyethylene cages with wood-shaving bedding and had unrestricted access to commercial rodent diet (Nuvilab^®^) and filtered water throughout the study period.

### 2.3. Experimental Design

*A priori* sample size calculation was performed considering bone elemental composition assessed by SEM/EDS (calcium and phosphorus percentages) as the primary endpoint. The estimation was based on previous experimental studies conducted by our group involving alveolar bone alterations under inflammatory and osteopenic conditions [20,22]. A large effect size assumption (partial η^2^ ≥ 0.14), according to Cohen’s conventions, was adopted, considering α = 0.05 and statistical power (1 − β) of 80% for two-way ANOVA comparisons. Based on these assumptions, a minimum of five animals per group was estimated to be sufficient. To compensate for possible experimental losses, six animals were included in each group. Statistical assumptions and analyses were performed using GraphPad Prism version 8.00 (GraphPad Software, San Diego, CA, USA). Thus, the study followed a completely randomized design, with mice randomly allocated into four groups (n = 6 animals per group): sham-operated group treated with saline (Sham), ovariectomized group treated with saline (OVX), sham-operated group treated with HAs (Sham + HA), and ovariectomized group treated with HAs (OVX + HA).

HAs were administered at a dose of 80 mg/kg daily for 28 days, beginning two weeks after surgical recovery. The selected dosage and duration of treatment were based on previous studies conducted by our group using periodontitis and estrogen-deficiency models, in which significant modulation of bone and oxidative parameters was observed after 28 days of HA administration [20,21,22,23]. At the end of the experimental period, the animals were euthanized, and the uterus and both hemimandibles were collected.

### 2.4. Protocol for Sham Surgery and Ovariectomy (OVX)

After a two-week acclimatization period, the animals were randomly allocated into two experimental groups: an ovariectomized group (OVX, n = 12) and a sham-operated control group (Sham, n = 12). The allocation procedure was designed to ensure comparable baseline body weights between groups, avoiding significant initial differences. In the OVX group, bilateral ovariectomy was performed following previously described protocols [21]. In the sham group, the same surgical procedures were carried out; however, the ovaries were exposed and left intact. All surgical procedures were performed under general anesthesia induced by ketamine (100 mg/kg) combined with xylazine (10 mg/kg).

### 2.5. Supplementation with Humic Acids (HAs)

Two weeks after the surgical procedures, the animals were randomly allocated into four experimental groups according to treatment (n = 6 per group): sham-operated receiving saline (Sham), ovariectomized receiving saline (OVX), sham-operated treated with humic acids (Sham + HA), and ovariectomized treated with humic acids (OVX + HA). The HA solutions were prepared daily by dissolving the compound in sterile saline. Administration was carried out once daily by oral gavage over a 28-day period. Animals in the Sham and OVX control groups received 0.3 mL of saline following the same regimen. At the end of the experimental period, the animals were anesthetized via intraperitoneal injection of xylazine hydrochloride (10 mg/kg) and ketamine hydrochloride (80 mg/kg). Euthanasia was subsequently performed by cardiac puncture. After confirmation of death, the uteri were excised and weighed using an analytical balance.

### 2.6. Analysis of the Elemental Composition and Topography of the Mandibular Bone Using Scanning Electron Microscopy Coupled with Energy-Dispersive X-Ray Spectroscopy (SEM/EDS)

After euthanasia, the left hemimandibles were immersed in hydrogen peroxide for 12 h to facilitate the removal of residual soft tissues and subsequently air-dried at room temperature for two weeks. To assess bone topography and identify potential morphological alterations, each specimen was mounted on an aluminum stub using double-sided conductive carbon tape and examined by scanning electron microscopy (SEM) (Vega 3 LMU, TESCAN, Brno-Kohoutovice, Czech Republic). Evaluations were performed on the buccal surface below the first mandibular molar, and images were acquired at a magnification of 500×. A qualitative assessment was performed considering the number of pores, as well as their area and diameter within a standardized region of interest (ROI) (60.59 × 10^3^ µm^2^) located below the first mandibular molar in the upper right quadrant, as previously described by our research group [24]. For pore area and pore diameter analyses, multiple measurements were obtained within the standardized region of interest (ROI) from each specimen. These measurements were considered technical morphometric assessments rather than independent biological replicates. Therefore, the biological sample size remained n = 6 animals per group. All analyses were conducted by a trained, calibrated, and blinded examiner. Measurements were obtained using ImageJ software, version 1.54 (National Institutes of Health, Bethesda, MD, USA).

The elemental composition of calcium (Ca) and phosphorus (P) was determined by energy-dispersive X-ray spectroscopy (EDS) using an X-MaxN detector (Oxford Instruments, Abingdon, UK) [24]. Spectra were acquired at an accelerating voltage of 20 kV with a working distance of 13 mm. Data analysis was performed using AZtec software, version 3.1 (Oxford Instruments), following a well-established protocol [22].

### 2.7. Quantitative Histomorphometric Assessment of Alveolar Bone Loss

After SEM/EDS analysis, the left hemimandibles were harvested, sectioned along the midline, and fixed in 10% buffered formalin for subsequent histological processing. The specimens were then immersed in an 10% ethylenediaminetetraacetic acid (EDTA) solution until complete decalcification was achieved. Thereafter, routine histological procedures were performed for slide preparation, as previously described [25]. Each specimen was subsequently used for the evaluation of alveolar bone loss (ABL) through histomorphometric analysis.

Serial sections approximately 5 µm thick were obtained and stained with hematoxylin and eosin (H&E) for examination under light microscopy. Histological images were captured using a digital camera (SC30 CMOS Color Camera for Light Microscopy, Olympus Optical do Brasil Ltd.a., São Paulo, SP, Brazil) coupled to a binocular microscope (Olympus CX31, Olympus Optical do Brasil Ltd.a., São Paulo, SP, Brazil). Images were acquired using a 10× objective lens, and measurements were performed with ImageJ image analysis software version 1.54 (National Institutes of Health, Bethesda, MD, USA). For the histomorphometric assessment, three sections per animal were selected and photographed. The digitized images were subsequently transferred for histometric analysis.

All measurements of alveolar bone loss were conducted by a trained and blinded examiner who was unaware of the experimental group allocation. The distance between the epithelial junction (CEJ) and the alveolar bone crest (ABC), measured in the interproximal region between the first and second molars, was used as the primary outcome, following a previously established protocol [25].

### 2.8. Immunohistochemical Analysis of RANKL and OPG Expression in the Alveolar Bone

Right hemimandibles, previously processed and embedded in paraffin, were sectioned at a thickness of 4 µm and mounted on silanized glass slides for immunohistochemical analysis. Immunohistochemistry was performed following deparaffinization and rehydration of the samples. Endogenous peroxidase activity was initially blocked using a 3% H_2_O_2_ solution in methanol for 15 min. Subsequently, nonspecific binding sites were blocked for 30 min.

Subsequently, the sections were incubated for 60 min in a dark chamber at 37 °C with primary antibodies against RANKL (goat polyclonal, 1:100 dilution; Abcam, Cambridge, UK) and OPG (rabbit polyclonal, 1:100 dilution; Santa Cruz Biotechnology, Dallas, TX, USA), according to the manufacturer’s optimized protocol (DAKO HercepTest™ Immunohistochemistry Kit; Dako Denmark A/S, Glostrup, Denmark, 2020). After washing in phosphate-buffered saline (PBS) for 5 min, biotinylated secondary antibodies (linker) were applied, followed by incubation with streptavidin–horseradish peroxidase (HRP) conjugate for 60 min at room temperature. Finally, chromogenic detection was performed using 3,3′-diaminobenzidine (DAB). Counterstaining with Harris hematoxylin was carried out to facilitate visualization of cell nuclei.

Images were captured at magnifications of 4×, 10×, and 40× using a SC30 CMOS color camera coupled to an Olympus CX31 binocular microscope (Olympus Optical do Brasil Ltd.a., São Paulo, SP, Brazil), focusing on a standardized region of interest located between the first and second molars. Positively stained cells were quantified using ImageJ software (version 1.54f; National Institutes of Health, Bethesda, MD, USA) with the multipoint selection tool by a trained investigator blinded to sample identity. The RANKL:OPG ratio was calculated according to a previously validated protocol [26].

### 2.9. Tartrate-Resistant Acid Phosphatase (TRAP) Staining for Osteoclast Quantification in Alveolar Bone

Tartrate-resistant acid phosphatase (TRAP) staining was performed on silanized slides containing paraffin-embedded sections of right hemimandibles using a commercial kit (TRAP staining kit, Sigma-Aldrich, catalog no. 387A, St. Louis, MO, USA), following the manufacturer’s validated protocol [27]. Thus, 4 µm sections, after deparaffinization and rehydration, were immersed in a 50% acetone/alcohol solution for 1 min. Subsequently, in a dark chamber, the TRAP solution was applied to the sections, and the material was incubated at 37 °C for 1 h and 30 min.

The TRAP solution contained naphthol phosphate as substrate, Fast Red Violet LB salt, dimethylformamide (C_3_H_7_ON), and an acetate buffer. The acetate buffer was prepared from acetic acid, sodium acetate, tartrate, and distilled water. Finally, counterstaining with hematoxylin was performed for 1 min to allow nuclear visualization. This procedure resulted in a characteristic reddish-brown cytoplasmic staining in TRAP-positive multinucleated osteoclasts located along bone surfaces. Images were captured at magnifications of 4×, 10×, and 40× using an SC30 CMOS color camera coupled to an Olympus CX31 binocular microscope (Olympus Optical do Brasil Ltd.a., São Paulo, SP, Brazil), focusing on a standardized region of interest between the first and second molars for each animal across all experimental groups.

TRAP-positive osteoclasts were quantified by a trained investigator blinded to group allocation using ImageJ (version 1.54f; National Institutes of Health, USA) with the multipoint selection tool. Cell counts per mm^2^ of bone surface were tabulated in Excel and statistically compared among groups using appropriate tests.

### 2.10. Statistical Analyses

The data were analyzed using analysis of variance (ANOVA) with an F-test within a 2 × 2 factorial design (with HA treatment and OVX considered as factors) to assess group differences and their interaction (*p* < 0.05). For pore area and pore diameter analyses, mean values obtained from multiple measurements within the standardized ROI of each specimen were calculated and considered as biological replicates for statistical comparisons. When significant interaction effects were detected, Bonferroni’s post hoc test was applied (*p* < 0.05). All statistical analyses were performed using GraphPad Prism version 8.00 (GraphPad Software, San Diego, CA, USA).

## 3. Results

### 3.1. Confirmation of Estrogen Deficiency Following Ovariectomy

Successful establishment of estrogen deficiency following ovariectomy was confirmed by the significant reduction in uterine weight observed at euthanasia. Ovariectomized animals exhibited significantly lower uterine mass compared with Sham-operated groups (OVX: 0.0188 ± 0.0169 g; OVX + HA: 0.0212 ± 0.0193 g vs. Sham: 0.0461 ± 0.0053 g; Sham + HA: 0.0520 ± 0.0143 g; two-way ANOVA, main effect of surgery: *p* < 0.0001). No significant effects of HA treatment or interaction between surgery and treatment were detected (*p* > 0.05).

### 3.2. Elemental Composition and Topography of Mandibular Alveolar Bone Assessed by SEM/EDS

**Figure 1:**
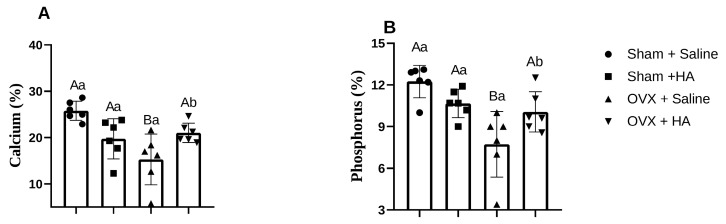
Percentages of calcium (**A**) and phosphorus (**B**) on the alveolar bone surface of ovariectomized (OVX) mice treated or not with humic acids (HAs). Bars represent mean ± SD, and symbols represent individual animals. Uppercase letters indicate significant differences between Sham and OVX groups, whereas lowercase letters denote significant differences between saline- and HA-treated animals within the same experimental condition (*p* < 0.05). Data were analyzed using two-way ANOVA followed by Bonferroni’s post hoc test (n = 6 per group).

The percentages of calcium and phosphorus on the alveolar bone surface were lower in the OVX group compared with the Sham group (*p* < 0.05, Figure 1A,B). However, ovariectomized mice treated with humic acids (HAs) exhibited higher levels of these elements than untreated OVX animals (*p* < 0.05). No significant differences were observed between the Sham and Sham + HA groups (*p* > 0.05) for either of the evaluated minerals.

**Figure 2:**
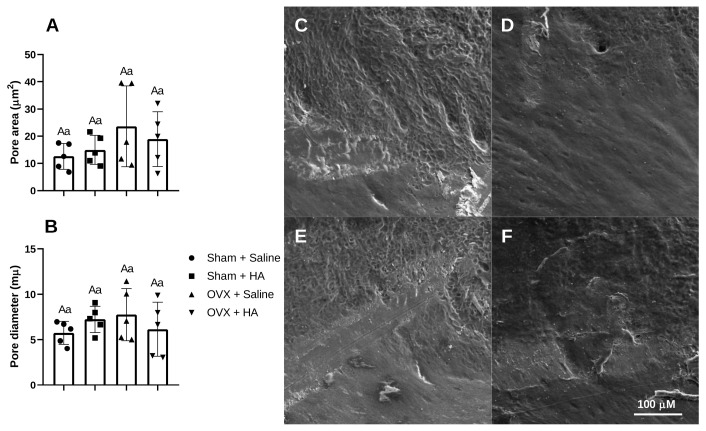
Quantitative and qualitative assessment of mandibular alveolar bone topography beneath the first molar in ovariectomized (OVX) mice treated with humic acids (HAs). (**A**) Pore area (µm^2^) and (**B**) pore diameter (µm) in the experimental groups. Bars represent mean ± SD, and symbols represent individual animals (n = 6 per group). No significant differences were observed among groups (two-way ANOVA, *p* > 0.05). (**C**–**F**) Representative scanning electron microscopy (SEM) images acquired at 500× magnification: (**C**) Sham-operated group treated with saline (Sham), (**D**) Sham-operated group treated with HAs (Sham + HA), (**E**) ovariectomized group treated with saline (OVX), and (**F**) ovariectomized group treated with HAs (OVX + HA). Scale bar: 100 µm. Uppercase letters indicate comparisons between Sham and OVX conditions, whereas lowercase letters indicate comparisons between treatments (saline vs. HA) within the same condition. Identical letters indicate the absence of statistically significant differences *(p* > 0.05).

No significant effects of OVX status, HA treatment, or OVX × HA interaction were observed for pore area or pore diameter measurements (*p* > 0.05; Figure 2A–F; Supplementary Table S1).

### 3.3. Alveolar Bone Resorption (ABR) Assessed by Histomorphometry

**Figure 3:**
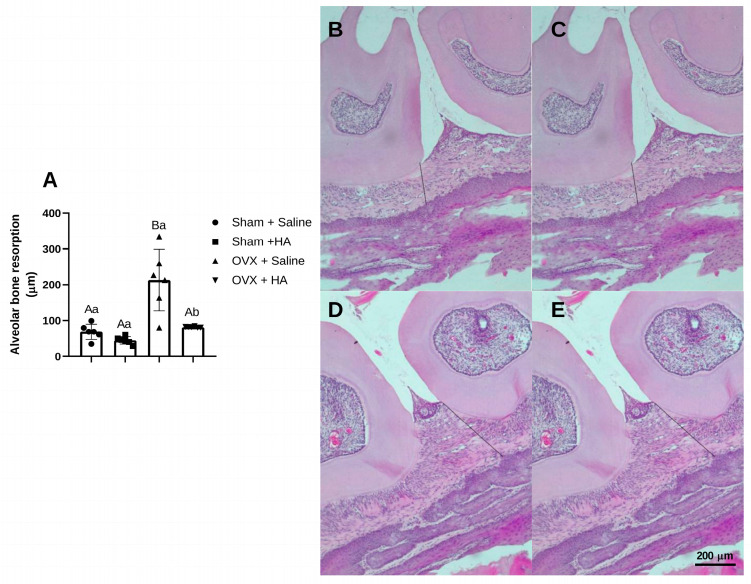
Assessment of mandibular alveolar bone in ovariectomized (OVX) mice treated with humic acids (HAs). (**A**) Alveolar bone resorption (µm) in the experimental groups. Bars represent mean ± SD, and symbols represent individual animals (n = 6 per group). Uppercase letters indicate significant differences between OVX and Sham groups (*p* < 0.05), whereas lowercase letters denote significant differences between HA-treated and untreated groups within the same experimental condition (*p* < 0.05). Data were analyzed using two-way ANOVA followed by Bonferroni’s post hoc test. (**B**–**E**) Representative histological images of the mandibular alveolar bone beneath the first molar: (**B**) Sham-operated group treated with saline (Sham), (**C**) Sham-operated group treated with HAs (Sham + HA), (**D**) ovariectomized group treated with saline (OVX), and (**E**) ovariectomized group treated with HAs (OVX + HA). Images were stained with hematoxylin and eosin (H&E).

Alveolar bone resorption, assessed by the distance between the alveolar bone crest and the epithelial insertion, was greater in the OVX group than in the Sham group (*p* < 0.05). Animals in the OVX + HA group exhibited lower levels of alveolar bone resorption compared with those in the OVX group (*p* < 0.05; Figure 3A). Figure 3B–E represent the alveolar bone resorption (ABR) assessed by histomorphometry among the experimental groups.

### 3.4. Immunolabeling of Receptor Activator of Nuclear Factor Kappa-B Ligand (RANKL) and Osteoprotegerin (OPG)

**Figure 4:**
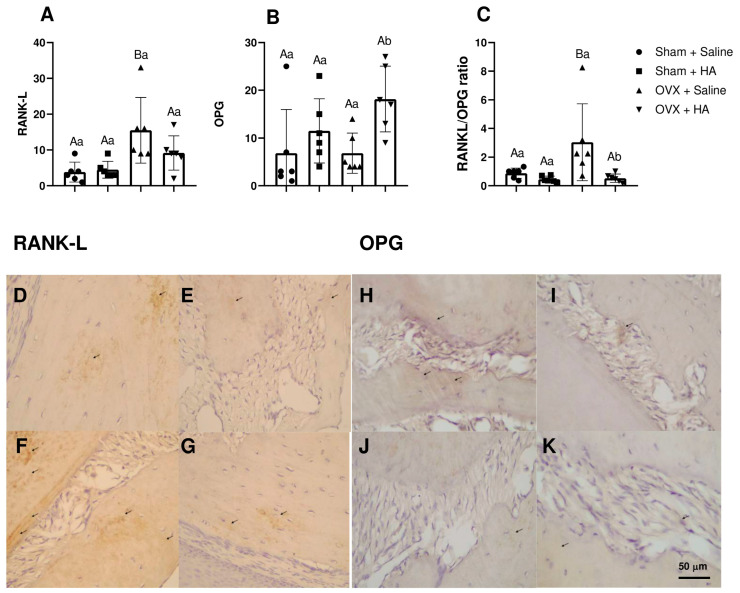
Immunolabeling of RANKL (**A**), OPG (**B**), and the RANKL/OPG ratio (**C**) in the mandibular alveolar bone of ovariectomized (OVX) mice treated or not with humic acids (HAs). Bars represent mean ± SD, and symbols represent individual animals. Uppercase letters indicate statistically significant differences between Sham and OVX groups, whereas lowercase letters denote significant differences between treatments (saline vs. HAs) within the same experimental condition (*p* < 0.05). Panels (**D**–**K**) present representative images of immunolabeling for RANKL (**D**–**G**) and OPG (**H**–**K**) across the experimental groups. Arrows indicate positively immunolabeled cells/areas identified by brownish staining in the cytoplasm and/or extracellular matrix along the bone surface. Data were analyzed using two-way ANOVA followed by Bonferroni’s post hoc test (n = 6 per group). Images were acquired at 40× magnification. Scale bar: 50 µm.

The number of RANKL-immunopositive cells was higher in the OVX group than in the Sham group (*p* < 0.05). No significant differences were observed for this parameter between the OVX and OVX + HA groups (*p* > 0.05; Figure 4A). Regarding OPG immunolabeling, no significant differences were detected between the OVX and Sham groups (*p* > 0.05). However, the number of OPG-immunopositive cells was greater in the OVX + HA group compared with the OVX group (*p* < 0.05; Figure 4B). The RANKL/OPG ratio was higher in the OVX group than in both the Sham and OVX + HA groups (*p* < 0.05).

### 3.5. Assessment of Osteoclasts by Tartrate-Resistant Acid Phosphatase (TRAP) Staining

**Figure 5:**
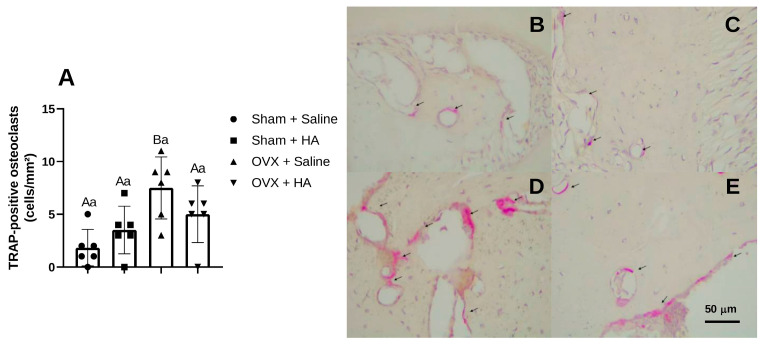
Quantification and representative photomicrographs of tartrate-resistant acid phosphatase (TRAP)-positive osteoclasts in the mandibular alveolar bone of Sham and ovariectomized (OVX) mice treated with saline or humic acids (HAs). (**A**) Number of TRAP-positive multinucleated osteoclasts per mm^2^ of bone surface. Bars represent mean ± SD, and symbols represent individual animals. Uppercase letters indicate significant differences between Sham and OVX groups, whereas lowercase letters indicate significant differences between treatments within the same experimental condition (*p* < 0.05). (**B**–**E**) Representative photomicrographs (40× magnification) of TRAP staining: (**B**) Sham + saline; (**C**) Sham + HA; (**D**) OVX + saline; and (**E**) OVX + HA. Black arrows indicate TRAP-positive multinucleated osteoclasts characterized by intense reddish staining in the cytoplasm and located along bone surfaces, particularly at resorption lacunae. Scale bar: 50 µm. Data were analyzed using two-way ANOVA followed by Bonferroni’s post hoc test (n = 6 per group).

The number of TRAP-positive cells was higher in the OVX group than in the Sham group (*p* < 0.05). No significant differences were observed between the OVX and OVX + HA groups for this parameter (*p* > 0.05; Figure 5A).

## 4. Discussion

Among the main findings of our study are the improvement in mandibular mineral composition and the attenuation of alveolar bone resorption in ovariectomized animals treated with HAs. Additionally, we observed a higher number of OPG-positive cells in HA-treated OVX animals, along with a reduced RANKL/OPG ratio compared with untreated counterparts. These findings reinforce the protective effects of HAs on alveolar bone, consistent with those previously demonstrated in an experimental model of periodontitis [20,22,28].

The ovariectomy model is well established in the literature as a method for inducing estrogen deficiency [29,30]. The cessation of ovarian estrogen production leads to, among other effects, a reduction in OPG expression, thereby allowing increased RANKL activity and subsequent osteoclast activation [5]. This process results in enhanced bone demineralization and increased bone resorption [5]. In the present study, animals in the OVX + saline group exhibited increased immunolabeling for RANKL, as well as a higher number of TRAP-positive osteoclasts compared to those in the Sham + saline group. Furthermore, the RANKL/OPG ratio, an established molecular indicator of bone resorption [31], was significantly elevated in ovariectomized animals relative to sham controls. These findings confirm the successful establishment of a high bone turnover and resorptive phenotype induced by estrogen deficiency [32].

Although most studies investigating estrogen deficiency have primarily focused on reductions in bone mineral density in long bones, there is a growing concern regarding alveolar bone [33,34]. With the progressive aging of the population, an increasing number of dental and orthodontic procedures are being performed in older individuals [35,36]. In this context, alveolar bone resorption may accelerate tooth loss, compromise implant stability, and negatively affect aesthetic outcomes [37]. Therefore, safe strategies to attenuate these effects are of considerable clinical interest [37]. In this context, the effects of HA treatment on alveolar bone have previously been described in ligature-induced periodontitis models, where these effects were associated with the anti-inflammatory and antioxidant properties of these compounds [20,22,28]. These properties are related to their complex molecular composition, characterized by phenolic, carboxylic, and quinone functional groups [38]. These structural features enable direct scavenging of reactive oxygen species (ROS), electron transfer reactions, and metal chelation, thereby reducing oxidative stress [38]. In addition, HAs modulate endogenous antioxidant defenses by increasing the activity of enzymes such as superoxide dismutase and catalase [39,40]. Their anti-inflammatory effects are mediated by the downregulation of pro-inflammatory cytokines and the inhibition of key signaling pathways such as NF-κB [18]. Furthermore, their amphiphilic structure allows interaction with cellular membranes and modulation of immune responses [41]. The characterization of the HAs used in the present study was previously described by our research group [20,21,22]. In general, HAs presented carbon and oxygen as the predominant elements, in addition to the absence of potentially toxic metals [21]. The atomic H/C ratio of 1.63 and O/C ratio of 0.62, together with elevated total and phenolic acidity, indicated the presence of oxygenated functional groups and high chemical reactivity [20]. Furthermore, 1H and 13C CP/MAS NMR analyses suggested a molecular structure composed of aliphatic, aromatic, and carbonyl groups, with predominance of hydrophobic domains and an aromaticity index of 28.28% [22]. Additionally, FTIR analysis confirmed the presence of hydroxyl, amine, amide, carboxylate, and aromatic groups, reinforcing the complex and highly functionalized nature of HAs.

To the best of our knowledge, this is the first study to investigate molecular markers of alveolar bone remodeling in a model of estrogen deficiency treated with HAs. Although periodontitis and estrogen deficiency-induced osteoporosis share mechanisms associated with bone resorption, particularly increased inflammatory activity and enhanced osteoclastogenesis, they differ in their primary etiological triggers [42]. While periodontitis is primarily driven by bacterial biofilm and local inflammation, estrogen deficiency induces a systemic imbalance in bone remodeling, characterized by increased osteoclast activity and altered RANKL/OPG signaling [43]. Despite these differences, the convergence of these pathways suggests that therapeutic strategies targeting inflammation and oxidative stress may be effective in both conditions [42,44].

In the present study, we observed that HA supplementation attenuated alveolar bone resorption and improved mineral content in ovariectomized animals. This effect may be attributed to increased OPG expression and a reduction in the RANKL/OPG ratio, as also observed in our results, indicating a shift toward an anti-resorptive signaling environment. Interestingly, although HA treatment attenuated alveolar bone resorption in OVX animals, the reduction in TRAP-positive osteoclast number was only partial and was not statistically significant. This finding suggests that the protective effects of HAs may not be exclusively related to suppression of osteoclast recruitment, but also to modulation of osteoclast activity and local bone remodeling dynamics. In this context, the increased OPG immunolabeling and reduced RANKL/OPG ratio observed in HA-treated animals support the hypothesis that HAs may influence osteoblast–osteoclast signaling and the local bone microenvironment, thereby attenuating bone resorptive activity even in the presence of TRAP-positive osteoclasts. OPG is a soluble decoy receptor that plays a central role in bone remodeling by binding to RANKL, thereby preventing its interaction with RANK on osteoclast precursors and inhibiting osteoclastogenesis [31]. The balance between RANKL and OPG is a key determinant of bone resorption, with an increased RANKL/OPG ratio being associated with enhanced osteoclastic activity and bone loss [43]. Antioxidant and anti-inflammatory agents, such as HAs, are thought to reduce ROS levels and inhibit NF-κB activation, leading to increased OPG expression and decreased osteoclast activity [23,45].

The preservation of mineral content in the alveolar bone of HA-treated OVX animals further supports this hypothesis. Mineral composition is a critical determinant of bone quality, and its maintenance indicates a protective effect against demineralization [46]. Interestingly, calcium and phosphorus levels in HA-treated OVX animals approached those observed in the Sham groups, suggesting partial preservation of alveolar bone mineral composition despite estrogen deficiency. One possible explanation for this finding may involve the antioxidant and anti-inflammatory properties previously described for humic acids [20,21,22,23,31]. Estrogen deficiency is known to increase oxidative stress and inflammatory mediators associated with enhanced osteoclastic activity and bone resorption. Therefore, attenuation of these processes by HAs may contribute to preservation of bone mineral content. Additionally, another factor potentially associated with the maintenance of bone composition is the chelating property of HAs [31]. Previous studies have shown that HAs may enhance gastrointestinal proteolytic and lipolytic enzyme activity, thereby influencing calcium and phosphorus absorption [31]. Increased proteolysis may increase the availability of free amino acids such as lysine and arginine, reducing the formation of insoluble calcium complexes and improving mineral bioavailability [12,32]. Furthermore, increased lipolytic activity may reduce the formation of insoluble calcium–fatty acid complexes that impair intestinal calcium absorption [31]. Together, these mechanisms may contribute to the preservation of mineral availability and alveolar bone composition in HA-treated ovariectomized animals. This interpretation is supported by the reduced RANKL/OPG ratio observed in HA-treated OVX animals, suggesting attenuation of the pro-resorptive microenvironment associated with estrogen deficiency. However, the present study was not designed to directly investigate the molecular mechanisms involved in these effects, and therefore, these interpretations should be considered speculative. Overall, these findings suggest that HAs exert a multifaceted effect on bone remodeling, acting primarily through the modulation of the bone microenvironment. Rather than directly suppressing osteoclast numbers, HAs appear to influence the regulatory balance between pro- and anti-resorptive factors, ultimately leading to reduced bone loss.

Despite these promising findings, our study has some limitations. First, the absence of gold-standard imaging techniques, such as micro-computed tomography, precludes a more detailed evaluation of bone microarchitecture. Additionally, bone remodeling mediators (RANKL and OPG) were assessed using semiquantitative techniques, limited to the surface of the evaluated bone. Thus, future studies should integrate molecular and imaging approaches to better elucidate the mechanisms underlying the effects of HAs on alveolar bone. Furthermore, complementary in vitro studies investigating the direct effects of HAs on osteoclastogenesis and bone remodeling-related signaling pathways, including the RANKL/OPG balance, would provide additional mechanistic insights into the biological effects of HAs. Additionally, the absence of serum estradiol assessment represents a limitation of the present study, although uterine weight reduction confirmed the successful establishment of estrogen deficiency following ovariectomy. Although the sample size was defined based on *a priori* power analysis for the primary endpoint, the relatively limited number of animals and the evaluation of only a single HA dosage may restrict broader conclusions regarding dose–response relationships and generalizability of the findings. Additionally, the absence of a positive control group using standard anti-resorptive therapies, such as 17β-estradiol, limits direct comparisons between HAs and established treatments for estrogen deficiency-induced bone loss. Therefore, future studies should investigate the effects of HAs in comparison with conventional anti-osteoporotic therapies. In addition, although significant effects on alveolar bone remodeling were observed after 28 days of treatment, longer experimental periods may provide a more comprehensive understanding of the long-term effects of HAs on bone metabolism and microarchitectural preservation. Therefore, future studies should investigate prolonged HA administration protocols exceeding 6 weeks.

## 5. Conclusions

Treatment with biomass-derived HAs attenuated alveolar bone loss associated with estrogen deficiency, in association with changes in the RANKL/OPG balance, particularly increased OPG expression. Overall, the present findings reinforce the potential protective effects of biomass-derived HAs against estrogen deficiency-induced alveolar bone loss and preserved bone mineral composition in ovariectomized mice, possibly through modulation of the RANKL/OPG balance and the bone remodeling microenvironment. However, the molecular mechanisms underlying these effects remain to be clarified.

## Data Availability

The raw data supporting the conclusions of this article will be made available by the authors on request.

## Supplementary Materials

The following supporting information can be downloaded at: https://www.mdpi.com/article/10.3390/biomedicines14061244/s1, Table S1: Detailed statistical outputs from two-way ANOVA analyses.
