# Supplementary material for: Humic Acid Therapy Mitigates Estrogen Deficiency-Induced Alveolar Bone Loss and Modulates the RANKL/OPG Balance
Source: Biomedicines. 2026 May 30;14(6):1244. doi: 10.3390/biomedicines14061244 (PMC13296437; doi:10.3390/biomedicines14061244)
Supplement: Supplementary file 1 [file biomedicines-14-01244-s001.zip › biomedicines-4320644-supplementary.pdf]

**Supplementary Table S1.** Detailed statistical outputs from two-way ANOVA analyses.

| Outcome | Effect | F(df1,df2) | Exact p-value |
|---|---|---|---|
| Calcium (%) | OVX | $F(1,17)=6.98$ | $p=0.017$ |
| | HA | $F(1,17)=0.02$ | $p=0.893$ |
| | OVX × HA | $F(1,17)=10.45$ | $p=0.005$ |
| Phosphorus (%) | OVX | $F(1,22)=4.72$ | $p=0.041$ |
| | HA | $F(1,22)=0.06$ | $p=0.811$ |
| | OVX × HA | $F(1,22)=0.90$ | $p=0.354$ |
| Pore area | OVX | $F(1,16) = 3.01$ | $p=0.102$ |
| | HA | $F(1,16)=0.07$ | $p=0.795$ |
| | OVX × HA | $F(1,16)=0.67$ | $p=0.426$ |
| Pore diameter | OVX | $F(1,16)=0.20$ | $p=0.661$ |
| | HA | $F(1,16)=0.004$ | $p=0.952$ |

| Outcome | Effect | F(df1,df2) | Exact p-value |
|---|---|---|---|
| | OVX × HA | F(1,16)=2.32 | p=0.148 |
| Alveolar bone resorption | OVX | F(1,20)=24.78 | p = 0.000072 |
| | HA | F(1,20)=18.04 | p = 0.000395 |
| | OVX × HA | F(1,20)=8.65 | p=0.008 |
| RANKL | OVX | F(1,20)=13.28 | p=0.002 |
| | HA | F(1,20)=1.60 | p=0.221 |
| | OVX × HA | F(1,20)=2.44 | p=0.134 |
| OPG | OVX | F(1,20)=1.37 | p=0.255 |
| | HA | F(1,20)=7.92 | p=0.011 |
| | OVX × HA | F(1,20)=1.37 | p=0.255 |
| RANKL/OPG ratio | OVX | F(1,20)=4.05 | p=0.058 |
| | HA | F(1,20)=6.93 | p=0.016 |
| | OVX × HA | F(1,20)=3.53 | p=0.075 |

| Outcome | Effect | F(df1,df2) | Exact p-value |
|---|---|---|---|
| TRAP-positive cells | OVX | F(1,20)=12.86 | p=0.002 |
| | HA | F(1,20)=0.17 | p=0.681 |
| | OVX × HA | F(1,20)=4.35 | p=0.050 |
